# Respectful maternity care among women who gave birth at public hospitals in Hadiya Zone, Southern Ethiopia

**DOI:** 10.3389/fpubh.2022.949943

**Published:** 2022-09-27

**Authors:** Tilahun Mengistie, Teshale Mulatu, Afework Alemayehu, Tesfaye Assebe Yadeta, Merga Dheresa

**Affiliations:** ^1^Department of Midwifery, Wochamo University College of Health Science, Hossana, Ethiopia; ^2^School of Nursing and Midwifery, College of Health and Medical Science, Haramaya University, Harar, Ethiopia

**Keywords:** respectful maternity care, childbirth, public hospitals, Hadiya Zone, Southern Ethiopia

## Abstract

**Background:**

A compassionate and respectful care during pregnancy and childbirth is one of the essential components of safe motherhood. However, most of the women in developing countries experience disrespectful and abusive maternity care during childbirth. Hence, this study assessed the status of respectful maternity care and associated factors to bridge the gap.

**Methodology:**

Facility-based cross-sectional study was conducted among mothers who delivered in public Hospitals in the Hadiya Zone, South Ethiopia from March 01 to 30, 2020. Data were collected using a pretested questionnaire through face-to-face interviews. Descriptive statistics was computed and multivariable logistic regression was fitted to identify predictors. Adjusted Odds Ratio (AOR) with 95% Confidence Interval was used to show the strength of association and level of significance was declared at *P*-value < 0.05.

**Result:**

This study showed that 67.8 % (95% CI: 62.4–70.8%) of mothers received respectful maternal care. Being married [AOR: 2.17, 95% CI (1.03–6.93)], Cesarean section delivery [AOR: 2.48, 95% CI (1.03–5.97)], and absence of complications during child birth [AOR: 4.37, 95% CI (1.41–13.56)], were significantly associated with respectful maternity care.

**Conclusions:**

The level of RMC in this study was moderate. Being married, Cesarean section delivery, and absence of complications during child birth were identified predictors of respectful maternity care. Therefore, tailored interventions aimed at improving respectful maternity care should target unmarried women, and women with complications of labor regardless of mode of delivery.

## Background

Pregnancy and childbirth are critical events in the reproductive life of women and denote a period of high susceptibility. Thus, compassionate and respectful care should be given for all pregnant women during labor and child birth to promote safe motherhood. Respectful maternity care refers to harmonized care given to all women to the highest possible standard and safeguards them from harm and mistreatment during labor and childbirth (1, 2).

Globally, averting maternal mortality has remained unfinished agenda of sustainable development goals (SDG) (3, 4). Despite the tremendous effort to reduce maternal mortality, the rate is still as high as 239 per 100,000 live births in low-income countries. Furthermore, MMR in Ethiopia is still 412/per 100,000 live births. This figure is very far from the target under the SDG by 2030, which is <70/100,000 (5). Even though skilled birth attendance (SBA) prevents a toll of maternal death, about 75% of mothers do not deliver in health institution in developing countries (5, 6).

Disrespectful and abusive maternity care is one of the main reasons for not utilizing maternal health services. Mistreatment and humiliation during labor and delivery can hinder women from accessing maternal health care for subsequent deliveries (7, 8).

Despite the negative effect of disrespectful and abusive care on the use of skilled birth care, there is currently no international consensus on how disrespect and abuse should be scientifically defined and measured (9, 10). In the perspective of this gap, Bowser and Hill identified seven categories of traits that describe disrespectful and abusive care during facility based child birth. The categories are physical abuse, non-consented care, non-confidential care, non-dignified care, discrimination, abandonment/neglect of care, and detention in facilities until hospital bills are paid (11).

Studies in Ethiopia portrayed the prevalence of disrespectful and abusive care ranging from 21.1 to 98.9% and the pooled prevalence found to be 49.4% (12–14), which are unacceptably high levels of obstetric violence and mistreatment.

The Ethiopian government in its 5-year health sector transformation plan tailored compassionate and respectful maternity care incorporating it in maternal health services packages (15). Although such investment was in place to alleviate this burning problem, their implementation in the national context is not promising.

Assessment of respectful maternity care during facility-based childbirth is necessary for the design, monitoring, and evaluation of interventions to promote respectful care during childbirth, especially in low-resource settings. In Ethiopia, few studies were conducted and evidences on the status of respectful maternity care and associated factors is limited. Therefore, this study assessed the status of respectful maternity care and associated factors in public Hospitals, in Hadiya Zone, Southern Ethiopia.

## Methods and materials

### Study area and period

The study was conducted at public hospitals in the Hadiya Zone, Southern Ethiopia. The zone has one teaching and referral hospital, two primary hospitals, 72 public health centers, 311 health posts, and 142 private clinics (16). The study was conducted from March 01 to 30/2020.

### Study design and population

A hospital based cross-sectional study was conducted among all mothers who gave birth in Hadiya Zone Public Hospitals during the study period. Seriously ill women who were unable to respond and who were referred from other health facilities after the second stage of labor were excluded from the study.

### Sample size and sampling procedure

The sample size was determined by using the double population proportion formula considering factors that are significantly associated with the outcome variable at (*p* < 0.05), a two-sided confidence level of 95%, a margin of error of 5%, power of 80%, and the ratio of exposed to unexposed 1:1 using Epi-calc statistical software. Taking the average monthly income as an exposure variable: outcome among exposed (79.6%) and among unexposed (67%) (17), and adding a 10% non-response rate the final sample size was 460.

All public hospitals were included in the study. Sample size was allocated to each hospital proportionally by considering the average number of attendants. A systematic random sampling was applied to select study participants using delivery registration logbook as a sampling frame.

### Data collection tool and procedure

A pre tested and structured questionnaires were prepared by reviewing literatures pertinent to the topic (7, 18–20). The tool contains socio demographic characteristics, obstetric characteristics of the participants and categories of RMC that women will get during facility based child birth. The status of RMC was measured using a validated tool for assessing RMC which was adapted from the Maternal and Child Health Integrated Program (18). Data were collected through an exit interview during discharge. Each eligible woman was approached privately in a separate room within the hospital environment. The data were collected by eight health professionals working outside the study Hospitals. A 2-day training was given to both the data collectors and the supervisors regarding the objective of the study, data collection tools, and ways of data collection. The collected data was checked by supervisors for its completeness and consistency daily.

### Data processing and analysis

Data were entered into the computer using Epi-data version 4.2 and exported to SPSS version 23 for analysis. Bi-variable analysis was carried out to see the association of each of the independent variables with the outcome variable (Respectful maternity care). Hosmer and Lemeshow's goodness-of-fit test was used to assess whether the necessary assumptions were fulfilled. All variables with a *p*-value ≤0.2 were taken into the multivariable model to control for all possible confounders. Finally, the results of multivariable logistic regression analysis were presented in the adjusted odds ratio with 95% confidence intervals. The level of statistical significance was declared at *p*-value < 0.05.

### Measurements

In this study, women were considered to have respectful maternity care during labor and childbirth if they answered yes to all of those questions assessing RMC or verification criteria used for assessing the seven categories (performance standards) of RMC during labor and childbirth (19–22).

Women were considered as experienced disrespect and abuse if they answered no to one or more of those questions assessing RMC or verification criteria used for assessing the seven categories of RMC (18, 23, 24).

## Results

### Socio-demographic characteristics of the respondents

A total of 451 mothers participated in the study; which yields a response rate of 98.04%. Among mothers, 178 (39.1%) of the study participants were within the age group of 25–29 years. The mean age of the mothers was 28.6 (SD ± 4.04) years.

Concerning their marital status and residency, 398 (88.2) of them were married and 237 (52.1%) of the respondents were from urban. Of the total 159 (35.3%) of them attended primary education and 203 (45%) are housewives (Table 1).

**Table 1:** Sociodemographic characteristics on respectful maternity care among mothers who gave birth at Hadiya Zone public hospitals Southern Ethiopia, 2020 (*n* = 451).

**Variables**	**Category**	**Frequency (%)**
Maternal age	15–19	36 (7.9)
	20–24	142 (31.2)
	25–29	178 (39.1)
	30–35	76 (16.9)
	>35	19 (4.2)
Residence	Urban	214 (47.9%)
	Rural	237 (52.1%)
Marital status	Single	28 (6.2)
	Married	398 (88.2)
	^*^Others	25 (4.5)
Religion	Protestant	319 (70.7)
	Orthodox	91 (20.2)
	Muslim	35 (7.8)
	^**^Others	6 (1.3)
Ethnicity	Hadiya	280 (62.1)
	Kambata	88 (19.5%)
	Siltie	26 (5.8 %)
	Gurage	36 (8%)
	^***^Others	21 (4.6%)
Educational status	No formal education	103 (22.8%)
	Primary education	159 (35.3%)
	Secondary education	81 (18%)
	College and above	108 (23.9%)
Occupation	House wife	203 (45.01%)
	Government employee	100 (22.2%
	Private employee	68 (15.1%)
	Merchant	58 (12.8%)
	^****^Others	22 (4.8%)
Average monthly income	< 2,000 birr	152 (33.6)
	2,001–3,000 birr	130 (29.2)
	3,001–4,000	113 (24.8)
	4,001–5,000	34 (7.5)
	>5,000 birr	22 (4.8)

Others = ^*^divorced, widowed, ^**^catholic, pagans, etc., ^***^Amhara, Oromo, Tigre etc., ^****^daily laborer, housemaid etc.

### Obstetric characteristics of respondents

Of the total respondents, 439 (97.3.0%) had antenatal care follow up and 234 (51.4%) had two or more ANC visits. Regarding the gravidity of participants, 216 (47.5%) were multigravida. More than half 240 (53.2%) gave birth vaginally while 41 (14.4%) gave birth by caesarian section (Table 2).

**Table 2:** Provider related and obstetric characteristics of the respondents among mothers who gave birth at Hadiya Zone public hospitals Southern Ethiopia, 2020 (*n* = 451).

**Variables**	**Category**	**Frequency (%)**
ANC follow up	Yes	439 (97.3%
	No	12 (2.7)
ANC visits	< 2 times	144 (32.1%)
	2–4 time	253 (56.1%)
	>4 times	54 (11.9%
Parity	Only one	216 (47.8%)
	Two	153 (33.9%)
	Three	51 (11.3%)
	Four and above	31 (6.8%)
The profession of HCP	Midwife	347 (76.9%)
	Doctor	80 (17.7%)
	Others	24 (5.3%)
Sex of health care provider	Female	108 (23.9%)
	Male	343 (76.1%)

ANC, Antenatal care; HCP, Health care provider.

### Status of respectful maternity care

Overall, 67.8% (95%; CI: 62.4, 70.8%) of the women received respectful maternity care. Out of all participants, 41.9% didn't receive confidential care and 43.7% complained that HCPs (health care providers) did not take informed consent before any procedure (Table 3).

**Table 3:** Respectful maternity care categories among mothers who gave birth at Hadiya Zone public hospitals, Southern Ethiopia, 2020 (*n* = 451).

	**RMC**	
**Categories**	**Yes (%)**	**No (%)**
**Abuse free care**		
Never used physical force/abrasive behavior with the woman	409 (80.6%)	42 (19.4%)
Never physically restrains woman	424 (82.1%)	27 (17.9%)
Never separates woman from her baby unless	430 (85.3%)	21 (14.7%)
Provides comfort/pain-relief as necessary	397 (88.3%)	54 (11.7%)
Does not deny food or fluid to women in labor	430 (85.3%)	21 (14.7%)
**Total**	**82.6%**	**17.4%**
**Confidential care**		
Never discussed private information in a way that others could hear	364 (70.7%)	87 (29.3%)
Used drapes/covering/screen appropriately to protect woman's privacy not to be seen by other people (apart from health providers) during delivery	341 (65.6%)	110 (34.4%)
**Total**	**58.1%**	**41.9%**
**Informed consent**		
Great and introduces self to woman and companion	327 (62.5%)	124 (37.5%)
Encourage the women and companion to ask questions	329 (62.9%)	122 (37.1%)
Respond the women's question with politeness and truthfulness	366 (71.2%)	85 (28.8%)
The provider explains what was being done and what to expect throughout labor and delivery	340 (55.4%)	111 (44.6%)
the provider gives periodic updates on status and progress of labor	351 (67.8%)	100 (32.2%)
The provider allows the women to choose birth position as she wants	347 (66.9%)	104 (33.1%)
The provider obtains permission/consent prior to any procedure	349 (67.4%)	102 (32.6%)
**Total**	**56.3%**	**43.7%**
**Dignified care**		
The health care provider speaks politely to woman and her companion	401 (68.9%)	50 (31.1%)
Allows woman and her companion to observe cultural practices as much as possible	417 (72.5%)	34 (27.5%)
Never makes insults, intimidation, threats, shouted at, scolded, laughed, scorned or coerces woman or her companion	425 (84.2%)	26 (15.8%)
**Total**	**75.2%**	**24.8%**
**Abandonment-free care**		
The health care provider never ignored or abandoned the woman when she called for help	416 (72.2%)	35 (27.8%)
The provider never leaves woman alone or unattended during on the delivery couch	434 (76.2%)	17 (23.8%)
**Total**	**74.2%**	**25.8%**
**Free of discrimination**		
Speaks to the woman in a language and at a language-level that she understands	432 (78%)	19 (22%)
The health care provider didn't discriminate the women based on any specific attribute	414 (74%)	27 (26%**)**
**Total**	**76%**	**24%**
**Never detained or confined against her will**		
Facility doesn't have a policy to detain women who don't pay.	434 (86.2%)	17 (13.8%)
The women don't been forced to stay against their will	420 (75.3%)	31 (24.7%)
**Total**	**80.7%**	**19.3%**
**Overall self-reported respectful maternity care**	**Yes (67.8%)**	**No (32.2%)**

### Factors associated with respectful maternity care

The bivariable analysis result showed that marital status, educational status, parity, the profession of health care provider, sex of health care provider, mode of delivery, birth outcome, length of hospital stay, complications during labor, and delivery were significantly associated with respectful maternity care. After adjusting for other variables; marital status, mode of delivery, and complications during labor and delivery remained significantly associated with respectful maternity care in multivariable logistic regression.

Married women were two times more likely to obtain respectful maternity care than single women [AOR: 2.17 (1.03–6.93)]. The odds of respectful maternity care were 2.48 times higher among mothers who delivered by cesarean section compared to those who gave birth by spontaneous vaginal delivery [AOR: 2.485, 95%CI (1.03, 5.97)]. The odds of respectful maternity care were four times higher among those women who did not face complications during childbirth as compared to their counterparts [AOR: 4.37, 95% CI (1.41–13.56)] (Table 4).

**Table 4:** Factors associated with respectful maternity care among mothers who gave birth at public hospitals of Hadiya Zone, southern, Ethiopia 2020 (*n* = 451).

**Variable**	**Category**	**Respectful maternity care**		**COR (95% CI)**	**AOR (95% CI)**	***p*** **-value**
		**No**	**Yes**			
Monthly income	< 2,000	44 (28.8%)	109 (71.2%)	1	1	1
	2,001–3,000	48 (36.4%)	84 (63.6%)	0.706 (0.43–1.16)	0.560 (0.265–1.181)	0.914
	3,001–4,000	41 (36.3%)	72 (63.7%)	0.709 (0.422–1.191)	0.553 (0.241–1.271)	0.128
	4,001–5,000	8 (24.2%)	25 (75.8%)	1.261 (0.529–3.01)	0.553 (0.241–1.271)	0.463
	>5,000	7 (38.9%)	11 (61.1%)	0.634 (0.23–1.74)	0.691 (0.161–2.964)	0.235
Marital status	Single	9 (32.1%)	19 (67.9%)	1	1	
	Married	124 (31.3%)	272 (68.7%)	**1.526 (1.164, 2.577)**	2.17 (1.031–6.933)*	**0.001**
	*Others	15 (60%)	10 (40%)	0.351 (0.116–1.062)	0.580 (0.154–2.180)	0.081
Residence	Rural	60 (28%)	154 (72%)	1	1	1
	Urban	88 (37.4%)	147 (62.6%	1.361 (0.92–2.03)	2.23 (0.96–4.32)	0.136
Educational status	Primary	17 (28.3%)	43 (71.7%)	1.763 (1.02–3.04)	0.98 (0.39–2.465)	0.079
	Secondary	33 (40.7%)	48 (59.3%)	0.821 (0.461–1.461)	0.53 (0.208–1.357)	0.551
	≥College	32 (24.2%)	100 (75.8%)	1.427 (0.73–2.795)	1.52 (0.53–4.35)	0.283
ANC visit	No	7 (58.3%)	5 (41.7%)	1	1	1
	Yes	141 (32.3%)	296 (67.7%)	2.939 (0.917–9.423)	0.615 (0.07–5.39)	0.792
Parity	Only 1	63 (41.7%)	88 (58.3%)	1	1	1
	2	51 (23.6%)	165 (76.4%)	2.316 (1.47–3.63)	1.005 (0.459–2.203)	0.059
	3	19 (37.3%)	32 (62.7%)	1.206 (0.63–2.317)	0.61 (0.22–1.69)	0.642
	4	11 (50%)	11 (50%)	0.716 (0.29–1.75)	0.79 (0.199–3.14)	0.188
	≥5	4 (44.4%)	5 (55.6%)	0.895 (0.23–3.466)	0.86 (0.09–8.137)	0.974
Profession of provider	Doctors	35 (43.8%)	45 (56.2%)	1	1	1
	Midwives	102 (29.5%)	245 (70.6%)	1.861 (1.13–3.06)	0.576 (0.22–1.48)	0.153
	**Others	11 (64.7%)	13 (67.3%)	0.848 (0.335–2.15)	0.31 (0.069–1.411)	0.861
Sex of HCP	Female	48 (44.4%)	60 (55.6%)	1	1	1
	Male	100 (29.5%)	241 (70.7%)	1.93 (1.235–3.01)	0.768 (0.328–1.802)	0.439
Type delivery	SVD	84 (25.2%)	249 (74.8%)	1	1	1
	Cesarean	51 (59.3%)	35 (40.7%)	**4.59 (2.80–7.54)**	2.48 (1.03–5.97)*	0.004*
	Instrumental	13 (43.3%)	17 (56.7%)	1.98 (0.856–4.58)	2.262 (0.24–21.33)	0.216
Birth outcome	Alive	120 (29.5%)	287 (70.5%)	1	1	1
	Dead	28 (66.7%)	14 (33.3%)	4.783 (2.43–9.40)	2.472 (0.757–8.067)	0.062
Stay at HF	< 12 h	88 (26.7%)	242 (73.3%)	1	1	1
	12–24 h	7 (38.9%)	11 (61.1%)	3.018 (1.85–4.92)	1.23 (0.494–3.076)	0.065
	>24 h	8 (53.3%)	7 (46.7%)	1.725 (0.61–4.86)	2.005 (0.25–16.007)	0.403
	>2 days	45 (52.3%)	41 (47.7%)	0.960 (0.32–2.88)	0.60 (0.048–7.63)	0.891
Delivery complication	Yes	53 (43.4%)	69 (56.6%)	1		1
	No	95 (29.1%)	232 (70.9%)	**1.876 (1.22–2.88)**	4.37 (1.41–13.56)*	0.000*

Significant at ^*^P < 0.05, 1 = constant, CI, Confidence Interval; COR, Crude odds Ratio; AOR, Adjusted Odds Ratio; HCP, Health care provider; HF, Health facility; Others = *divorced, widowed, **professionals like Nurses, Health officers.

## Discussion

A compassionate and respectful care during pregnancy and childbirth is one of the essential components of safe motherhood. In this study, the overall magnitude of respectful maternity care during labor and childbirth was 67.8 % (95% CI: 62.4–70.8%). On the other hand, about one-third of women (32.2%) reported disrespect and abusive (D&A) care during labor and childbirth which was unacceptably high.

The magnitude of RMC in this study is by far higher than the study conducted in Addis Ababa (21%) (20), Harar (38.4%) (21), West Shewa, Oromia region (35.8%) (22), and the study conducted in public Hospitals of Benishangul Gumuz Region, Ethiopia (12.6%) (23). However, this study finding is lower than the study finding from Tanzania (85%) (24) and Kenya (80%) (25). The difference could be due to study setting differences, methodological variation, participants' educational and socio-economic status, service quality, and the ability of participants to report disrespect and abusive care.

This study revealed that married women were two times more likely to receive respectful maternity care compared to those who are not in a marital union. This finding may be a result of companion, which indicates that providers are more likely to be cautious about how they act and speak to a client when a companion of the client is present. This finding is also supported by many other studies (17, 23, 25–27).

Furthermore, participants who gave birth by cesarean section were 2.48 times more likely to receive respectful maternity care compared to participants who gave birth by spontaneous vaginal delivery. The result is in contrast with the study done in Bahirdar, Northwest Ethiopia, and Addis Ababa, Ethiopia (17, 28). This could be justified by study setting differences, case flows, staff workload, and attitude of health care providers.

Unexpectedly, women who didn't face complications during childbirth were about four times to receive RMC as compared to those who faced complications during birth. This is aligned with other studies conducted in Ethiopia (17, 21, 28). This might be because those mothers who develop complications are admitted and stayed for an extended time and they might perceive poor quality of care provided by health professionals during their stay.

Finally, as a limitation this study was based on self-report, hence it was not possible to validate claims made by respondents in the course of questionnaire administration. There might be the possibility of underestimating disrespect and abusive care within short duration of data collection period due to its sensitive nature. To minimize this bias, the data were collected in a private room within the hospital setup.

## Conclusion

The level of respectful maternity care in this study was moderate in comparison with other studies in the country. But still, about one-third of women experienced disrespect and abusive care while they gave birth at a health facility. Therefore, concerned body and stakeholders should devise strategies to avoid disrespect and abusive care during labor and childbirth. Furthermore, due emphasis should be given for improving RMC among unmarried women, and women with complications of labor regardless of mode of delivery.

## Data availability statement

The original contributions presented in the study are included in the article/supplementary material, further inquiries can be directed to the corresponding author.

## Ethics statement

The studies involving human participants were reviewed and approved by Institutional Health Research Ethics Review Committee (IHRERC) of the College of Health and Medical Sciences, Haramaya University. The patients/participants provided their written informed consent to participate in this study.

## Author contributions

TMe drafted the study. TY and MD monitored the study process. TMu and AA was involved in report writing and drafted the manuscript. All authors have read and approved the final manuscript.

## Funding

This study was funded by Haramaya University. The funding organization has no role in designing the study, data collection, analysis, interpretation, protocol writing, and submission.

## Conflict of interest

The authors declare that the research was conducted in the absence of any commercial or financial relationships that could be construed as a potential conflict of interest.

## Publisher's note

All claims expressed in this article are solely those of the authors and do not necessarily represent those of their affiliated organizations, or those of the publisher, the editors and the reviewers. Any product that may be evaluated in this article, or claim that may be made by its manufacturer, is not guaranteed or endorsed by the publisher.

## Abbreviations

ANC: Antenatal Care
AOR: Adjusted Odds Ratio
CI: Confidence interval
D&A: Disrespect and Abuse
RMC: Respectful Maternity Care
MMR: Maternal Mortality Rate
SDG: Sustainable Development Goal
SBA: Skilled Birth Attendant.
